# Kinetic Features of 3′-5′ Exonuclease Activity of Human AP-Endonuclease APE1

**DOI:** 10.3390/molecules23092101

**Published:** 2018-08-21

**Authors:** Alexandra A. Kuznetsova, Olga S. Fedorova, Nikita A. Kuznetsov

**Affiliations:** 1 Institute of Chemical Biology and Fundamental Medicine (ICBFM), Siberian Branch of Russian Academy of Sciences, Novosibirsk 630090, Russia; sandra-k@niboch.nsc.ru; 2 Department of Natural Sciences, Novosibirsk State University, 2 Pirogova St., Novosibirsk 630090, Russia

**Keywords:** AP-endonuclease, DNA repair, exonuclease activity, pre-steady-state kinetics

## Abstract

Human apurinic/apyrimidinic (AP)-endonuclease APE1 is one of the key enzymes taking part in the repair of damage to DNA. The primary role of APE1 is the initiation of the repair of AP-sites by catalyzing the hydrolytic incision of the phosphodiester bond immediately 5′ to the damage. In addition to the AP-endonuclease activity, APE1 possesses 3′-5′ exonuclease activity, which presumably is responsible for cleaning up nonconventional 3′ ends that were generated as a result of DNA damage or as transition intermediates in DNA repair pathways. In this study, the kinetic mechanism of 3′-end nucleotide removal in the 3′-5′ exonuclease process catalyzed by APE1 was investigated under pre-steady-state conditions. DNA substrates were duplexes of deoxyribonucleotides with one 5′ dangling end and it contained a fluorescent 2-aminopurine residue at the 1st, 2nd, 4th, or 6th position from the 3′ end of the short oligonucleotide. The impact of the 3′-end nucleotide, which contained mismatched, undamaged bases or modified bases as well as an abasic site or phosphate group, on the efficiency of 3′-5′ exonuclease activity was determined. Kinetic data revealed that the rate-limiting step of 3′ nucleotide removal by APE1 in the 3′-5′ exonuclease process is the release of the detached nucleotide from the enzyme’s active site.

## 1. Introduction

**Figure 1:**
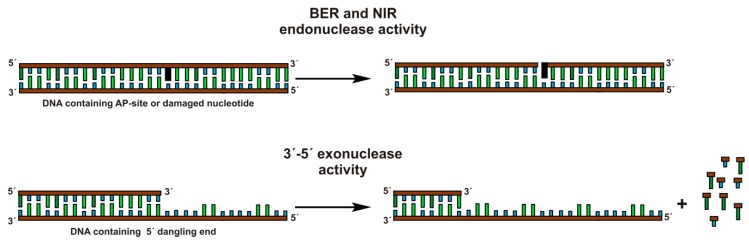
Types of catalytic activity for APE1.

Human apurinic/apyrimidinic endonuclease 1 (APE1), also known as a redox factor 1 (Ref-1) is a multifunctional enzyme [1]. The primary physiological function of APE1 is the incision of the phosphodiester bond immediately 5′ to an apurinic/apyrimidinic (AP) site, which generates a single-strand break with 5′-deoxyribose phosphate and 3′-hydroxyl ends (Figure 1) [2,3,4]. APE1 possesses some minor activities: 3′-5′ exonuclease, 3′-phosphodiesterase, 3′-phosphatase, and RNase H [5,6,7,8]. In addition to these activities, APE1 can recognize and convert some damaged or modified nucleotides such as 5,6-dihydrouridine [9], α-anomers [10], etheno derivatives [11,12], bulky photoadducts [13], benzene derivatives [14], and 2′-deoxyuridine [15]. This type of APE1 activity was named as the “nucleotide incision repair” (NIR) activity [16]. The heterogeneity of substrate specificity of the enzyme could be elucidated by structural data. Nonetheless, the currently available structural data mainly represent different variants of the free enzyme [17,18,19] and its complexes with nicked or abasic DNA [20,21,22]. Analysis of these data has shown that, for endonuclease activity, specific contacts in the complex APE1·DNA are formed, which result in the change of DNA conformation including eversion of an AP-site from the double helix. At the same time, the binding to DNA leads to slight structural rearrangements in the APE1 molecule itself. It has also been shown that amino acid residues of APE1 interact mainly with only one DNA chain, which contains an AP-site by forming hydrogen bonds and electrostatic contacts between DNA backbone phosphates and amino acid side groups or amide groups of peptide bonds. Recently, a set of APE1-DNA structural snapshots was reported revealing that APE1 removes 3′ mismatches and 3′-phosphoglycolate by placing the 3′ group within the intra-helical DNA cavity via a non-base-flipping mechanism [23]. It has been suggested that the hydrophobic pocket, which is composed of Phe-266 and Trp-280, plays a role in the substrate specificity [23,24]. The catalytic reaction is initiated by a nucleophilic attack of the oxygen atom of an H_2_O molecule coordinated directly or through an Mg^2+^ ion by Asp-210 on the 5′-phosphate group of the AP-site [20,22]. In alternative catalytic mechanisms [25,26], the role of the nucleophilic base plays the His-309 residue or the phenolate form of the Tyr-171 residue.

**Scheme 1:**
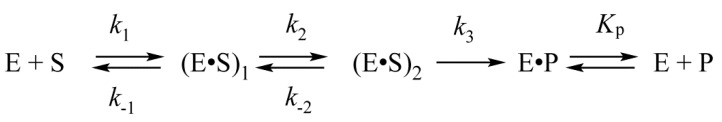
The kinetic mechanism of the interaction between APE1 and DNA containing an abasic site. Е is the enzyme, S is a DNA substrate, (E·S)_1_ and (E·S)_2_ are complexes of the enzyme with the substrate, Р is a product of substrate conversion, E·P is a complex of the enzyme with the product, *k*_i_ and *k*_−i_ denote rate constants of direct and reverse reactions (*i* = 1 or 2) of reversible steps, *k*_3_ is a rate constant of the catalytic reaction, and *K*_p_ is the equilibrium dissociation constant for the Е·Р complex.

In our earlier studies, kinetic analysis of conformational changes of the enzyme and of a DNA molecules has been performed on the endonuclease process catalyzed by APE1 [27,28,29]. For DNA substrates containing an AP-site or its synthetic analog 2-oxymethyl-3-oxy-tetrahydrofuran (F-site), the kinetic scheme containing two reversible steps of DNA-substrate binding has been determined (Scheme 1) [30,31]. It was hypothesized that the first binding step represents the formation of a nonspecific complex of APE1 with DNA: (E·S)_1_. In the course of the second step, when the (E·S)_2_ complex is produced, the amino acid residues and a Mg^2+^ ion in the active site of the enzyme form specific contacts with 5′ and 3′ phosphates and ribose residues of AP-DNA. During this step, the AP-site is everted to the active site of the enzyme so that a catalytically active conformation of APE1 is generated. After that, the irreversible chemical step of hydrolysis of the phosphodiester bond 5′ to the AP-site proceeds. The final, fourth step is the reversible release of product P, which contains a nick in one DNA chain from the complex with the enzyme E·P.

In spite of intensive studies regarding the features of the major endonuclease activity of APE1, the mechanisms of other activities of this enzyme toward DNA substrates with significantly different structures are still insufficiently examined. It was shown earlier that, under conditions optimal for AP endonuclease activity, the 3′-5′ exonuclease activity is six to seven orders of magnitude lower than the endonuclease activity [32]. Therefore, in the present work we were focused on the comparison of various DNA substrates in the course of only 3′-5′ exonuclease processing. For this purpose, a comparative kinetic analysis of the conformational changes of model DNA substrates in the course of their recognition and 3′-5′ exonuclease cleavage by APE1 was performed. By the stopped-flow fluorescence technique, changes in the fluorescence of 2-aminopurine residues introduced at various positions of the DNA substrate were recorded. The effects of undamaged and damaged 3′-end nucleotides or stability of the 3′-end pair on the 3′-5′ exonuclease activity were estimated. Taken together, the obtained data allowed us to elucidate kinetic features of the mechanism underlying the 3′-5′ exonuclease activity of APE1 and to conclude that the rate-limiting step is the release of the detached nucleotide from the active site of the enzyme.

## 2. Results and Discussion

**Figure 2:**
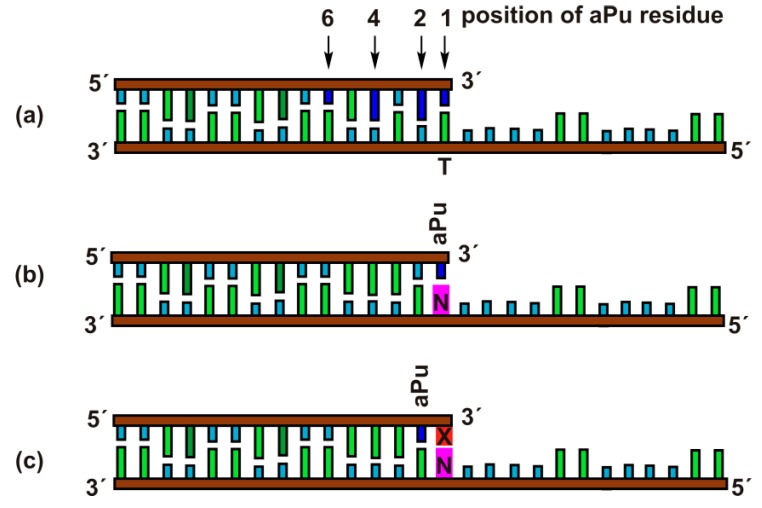
Schematic structures of DNA duplexes **Exo-aPu^j^/T** (**a**), **Exo-aPu^1^/N** (**b**), and **Exo-aPu^2^-X/N** (**c**) used as substrates and ligands of APE1.

Exonuclease activity of APE1 is effective toward duplexes containing gaps or 5′-dangling ends [32,33,34]. Therefore, for the kinetic analysis of the 3′-5′ exonuclease reaction, the duplexes of 15 and 28 nucleotides (nt) with a 5′-dangling end served as model DNA substrates (Figure 2). The 15 nt oligonucleotides contained the aPu residue at position 1, 2, 4, or 6 from the 3′ end in the duplexes **Exo-aPu^j^/T** (*j* = 1, 2, 4, and 6). The fluorescence of aPu is significantly quenched when this base is incorporated into single-stranded or double-stranded DNA [35,36,37]. This property of aPu enabled us to follow the rates of its removal from the different positions in a DNA chain. 

Moreover, using DNA substrates containing mismatched or damaged 3′ nucleotides allowed us to estimate the influence of the opposite-strand nucleotide (**Exo-aPu^1^/N**) and the structure of the 3′-neighboring nucleotide (**Exo-aPu^2^-X/N**) on the 3′-5′ exonuclease reaction.

### 2.1. The Position of aPu in the Duplex Affects APE1 3′-Exonuclease Efficiency

**Figure 3:**
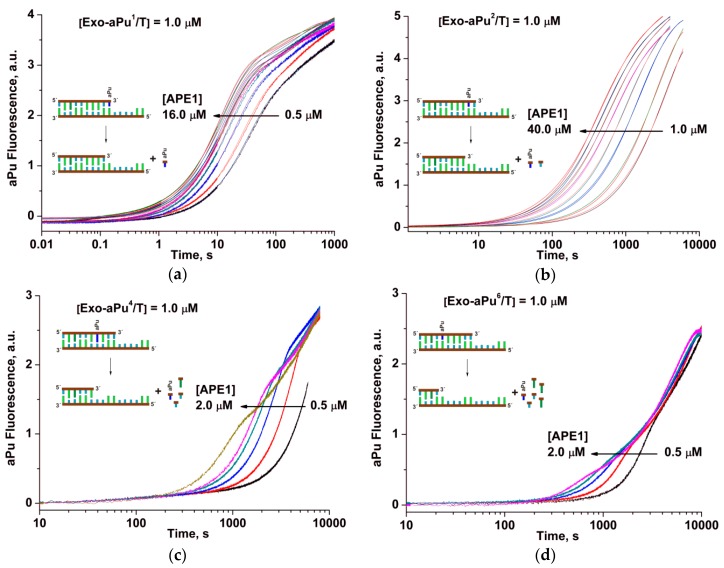
Changes in aPu fluorescence intensity during the interaction of APE1 with one of the substrates: (**a**) **Exo-aPu^1^/T**, (**b**) **Exo-aPu^2^/T**, (**c**) **Exo-aPu^4^/T**, or (**d**) **Exo-aPu^6^/T**. Concentrations of the enzyme and DNA are indicated in the panels. Kinetic traces for different APE1 concentrations are shown in different colors.

**Figure 4:**
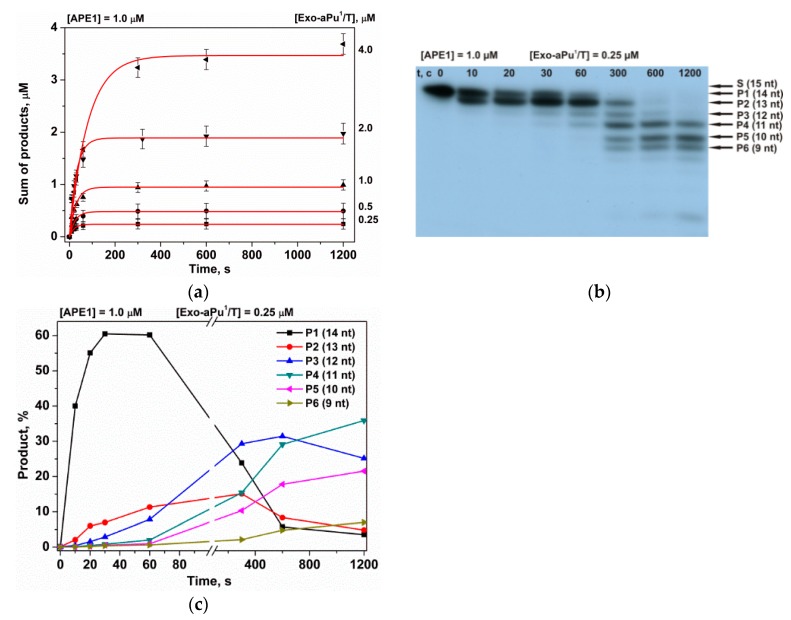
Kinetics of the accumulation of DNA products during an interaction with APE1 as detected by PAGE. (**a**) Accumulation of the total reaction products at various concentrations of substrate **Exo-aPu^1^/T**, (**b**) sequential formation of reaction products, and (**c**) the accumulation and consumption of intermediate truncated reaction products P*_n_* during sequential exonuclease removal of 3′-end nucleotides.

To determine the effect of a position of the aPu nucleotide in the duplex on the rate of its APE1-catalyzed removal, fluorescence kinetic traces were obtained for each of the duplexes **Exo-aPu^j^/T** at different concentrations of APE1 using the stopped-flow instrument (Figure 3). For all substrates, **Exo-aPu^j^/T**, the aPu fluorescence intensity increased with time, but the rate of this process decreased with the increase of the *j* value, i.e., with the moving of aPu away from the 3′ end of the short oligonucleotide. This result can be attributed to the process of sequential removal of non-fluorescent nucleotides beginning at the 3′ end until APE1 reaches the aPu nucleotide inside the sequence. To support this conclusion and find a possible correlation of the changes of aPu fluorescence intensity with the process of detachment of 3′ nucleotides, hydrolytic cleavage of 5′-^32^P-labeled 15 nt oligonucleotide of substrate **Exo-aPu^1^/T** was studied by polyacrylamide gel electrophoresis (PAGE, Figure 4). Fast accumulation of the total reaction products at various concentrations of substrate **Exo-aPu^1^/T** (Figure 4a) coincides with the time point of the aPu fluorescence intensity increase detected for this substrate. Moreover, as shown in Figure 4b,c, PAGE analysis allowed us to resolve the appearance and disappearance of shortened intermediate fragments of 5′-^32^P-labeled 15 nt oligonucleotide of substrate **Exo-aPu^1^/T** indicated as products P*_n_*, where *n* is the length of the fragment in nucleotides. Comparison of kinetic data obtained by fluorescence (Figure 3) and PAGE (Figure 4) analyses for substrates **Exo-aPu^j^/T** revealed that the phase of the increase in aPu fluorescence intensity coincided with the removal of the aPu nucleotide from the *j*th position of the 15 nt oligonucleotide.

**Figure 5:**
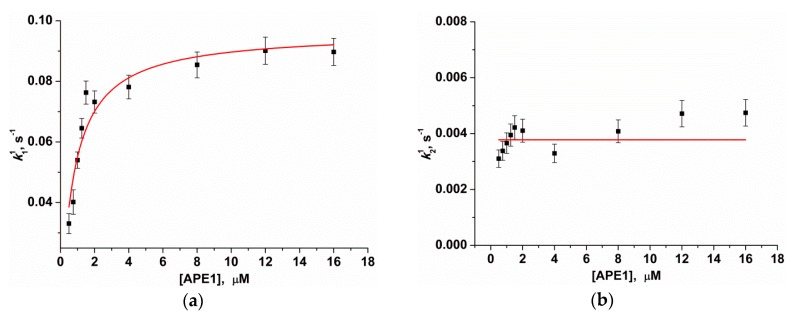
Dependences of the observed rate constants *k*_1_^1^ (**а**) and *k*_2_^1^ (**b**) on APE1 concentration. Data presented in (**a**) and (**b**) were fitted to a hyperbolic equation (Equation (1)) or to a linear function, respectively.

The kinetic parameters of the 3′-5′ exonuclease reaction—resulting in the removal of the 3′ aPu nucleotide from substrates **Exo-aPu^j^/T**—were calculated using Equation (3). Kinetic traces in Figure 2a characterizing the interaction of APE1 with substrate **Exo-aPu^1^/T** were best fitted by a two-exponent function (Equation (3), *i* = 2). The observed rate constant *k*_1_^1^ of the fast increase phase of aPu fluorescence intensity depends on the concentration of APE1 in a hyperbolic manner (Figure 5а). Hyperbolic dependence of *k*_1_^1^ indicates that the first phase includes a two-step kinetic mechanism. It is reasonable to suggest that the first step reflects a process of initial complex formation between the enzyme and substrate. Its conversion to catalytically active conformation includes the placement of a 3′ nucleotide in the enzyme’s active site. The second step in this mechanism could characterize the catalytic reaction of hydrolysis of the phosphodiester bond with the 3′-aPu nucleotide. Using Equation (1), the equilibrium constant of the binding of APE1 to substrate **Exo-aPu^1^/T** (*K*_bind_ = (1.4 ± 0.2) × 10^6^ М^−1^) as well as the rate constant of the catalytic reaction (*k*_detach_^aPu^ = 0.096 ± 0.004 s^−1^) were calculated.

*k*_1_^1^ = *K*_bind_ × [APE1] × *k*_detach_^aPu^/(*K*_bind_ × [APE1] + 1) (1)

Since the 3′-5′ exonuclease reaction proceeds with multiple turnover up to full substrate hydrolysis, it was likely that, after detachment of the aPu nucleotide from the 3′ end of the DNA chain by hydrolytic cleavage of the 3′-5′ phosphodiester bond, there was a step of removal of the detached nucleotides from the active site of the enzyme. The rate of the release of a detached aPu nucleotide could be associated with the network of contacts of this nucleotide with amino acid residues of the active site and most likely should not depend on the concentration of APE1. Calculated values of observed rate constants *k*_2_^1^ do not reveal their significant dependence on APE1 concentration (Figure 5b). Therefore, we hypothesized that the second phase of the growth of aPu fluorescence intensity represents the release of the detached aPu nucleotide from the active site of the enzyme. The linear approximation of this data allows us to calculate the average observed rate constant of aPu release *k*_2_^1^ = *k*_release_^aPu^ = 0.0038 ± 0.0002 s^−1^.

**Scheme 2:**
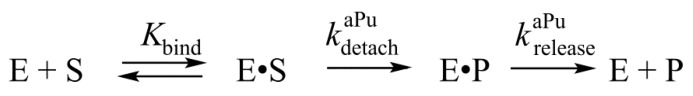
The kinetic mechanism of removal of 3′-aPu nucleotide. Е is the enzyme, S is a DNA substrate, E·S is a complex of the enzyme with the substrate, Р is a product of substrate conversion, E·P is a complex of the enzyme with the product, *K*_bind_ denotes the equilibrium constant of binding of APE1 to the substrate, *k*_detach_^aPu^ is a rate constant of the hydrolysis of the 3′-5′ phosphodiester bond, and *k*_release_^aPu^ represents the rate constant of the release of a detached aPu nucleotide from the active site of the enzyme.

Thus, the total kinetic mechanism of removal of 3′-aPu during the 3′-5′ exonuclease process is described in Scheme 2. The complex E·S in Scheme 2 corresponds to the catalytic complex of APE1 with a DNA substrate. The 3′-aPu detachment rate constants calculated by hyperbolic fitting were approximately 25-fold higher than the rate constant of the aPu nucleotide release from the active site of the enzyme, which indicates strong inhibition of APE1 by the first detached nucleotide. Recently published pre-steady-state quench-flow analysis of product accumulation in the course of 3′-5′ exonuclease reaction revealed a burst phase with a subsequent steady-state linear phase [23], which supports the notion that the rate-limiting step of enzyme turnover is the release of a detached nucleotide from the active site.

### 2.2. Determination of the Mean Rate of Removal of a 3′-End Nucleotide

**Figure 6:**
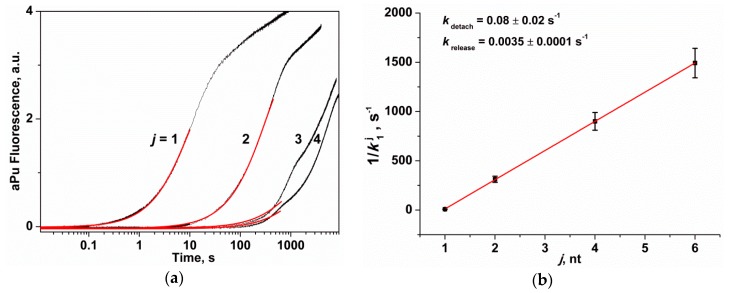
The change in the fluorescence intensity of aPu in the course of APE1 interaction with substrates **Exo-aPu^j^/T**. (**a**) Exponential analysis (red line) of the initial part of the kinetic curves (*j* is the position of the nucleotide relative to the 3′ end of the short oligonucleotide). [**Exo-aPu^j^/T**] = 1.0 μM, [APE1] = 2.0 μM. (**b**) Dependence of *k*_1_*^j^* on *j*, which is described by Equation (2).

In the course of interaction of APE1 with substrates **Exo-aPu^j^/T** (Figure 6a), initially the complex of the enzyme with the 3′-end nucleotide of the substrate is formed. Then the detachment and a subsequent release of the 3′-end nucleotide takes place and, afterward, the enzyme is shifted along the shortened DNA fragment to the second nucleotide and so far the *j*-th aPu residue would be detached. It could be proposed that the binding of the enzyme to a substrate—before the catalytic action and enzyme dislocation along DNA after catalysis—proceed faster than the catalytic reaction. Therefore, for substrates **Exo-aPu^2^/T**, **Exo-aPu^4^/T**, and **Exo-aPu^6^/T**, the time required for removal of a aPu nucleotide placed in the middle of the sequence is equal to the sum of the catalytic reaction periods required for the detachment of the 3′-end nucleotides and duration of the release of a detached nucleotide from the active site. Due to the disorder of the enzymatic process in the course of this repetitive multiple turnover process of the enzyme, the kinetic curves recorded for **Exo-aPu^2^/T**, **Exo-aPu^4^/T**, and **Exo-aPu^6^/T** were described with a multi-exponential function (Equation (3), *i* > 3). Therefore, we analyzed only the first exponential term in this equation, which contained the observed rate constant *k*_1_*^j^*. It is worth noting that the values of rate constants *k*_1_^1^ and *k*_1_^2^ (characterizing the removal of the first and the second nucleotide) differed approximately 35-fold. On the basis of the kinetic law for the rate of the sequential reactions [38], the following Equation (2) is true.

1/*k*_1_*^j^* = *j*/*k*_detach_ + (*j* − 1)/*k*_release_ = *j* × (1/*k*_detach_ + 1/*k*_release_) − 1/*k*_release_ (2)

where *j* is the position of aPu from the 3′ end, *k*_1_*^j^* is the observed rate constant of the first phase, *k*_detach_ is the mean rate constant of the catalytic reaction of hydrolysis of a 3′-5′ phosphodiester bond of any nucleotide at the 3′ end of the short oligonucleotide in DNA substrate, and *k*_release_ is the mean rate constant of a release of any detached nucleotide from the active site of APE1.

Based on Figure 6b, the reverse value of *k*_1_*^j^* linearly depends on *j*, which allows us to calculate the values of *k*_detach_ = 0.08 ± 0.02 s^−1^ and *k*_release_ = 0.0035 ± 0.0001 s^−1^ using Equation (2). Thus, these results reveal that the hydrolysis of a phosphodiester bond of any 3′-end nucleotide proceeds at least 10-fold faster than the release of this detached nucleotide from the active site of APE1. The rate constants of detachment and release of any nucleotide are in good agreement with the constants obtained for the aPu nucleotide by analysis involving a set of concentrations of substrate **Exo-aPu^1^/T** (*k*_detach_^aPu^ = 0.096 ± 0.004 s^−1^ and *k*_release_^aPu^ = 0.0038 ± 0.0002 s^−1^).

### 2.3. The Rate of the 3′-5′ Exonuclease Reaction Depends on Stability of the 3′-End Base Pair

**Figure 7:**
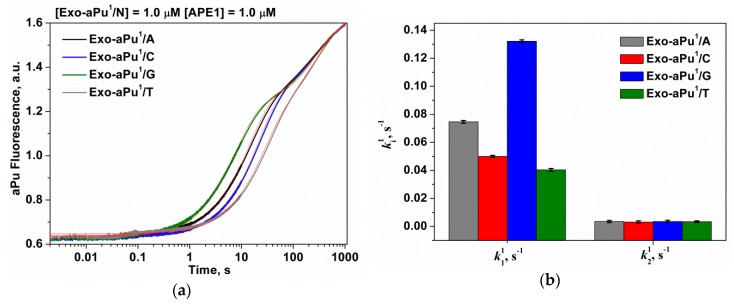
(**a**) A change in the intensity of aPu in the course of APE1 interaction with substrates **Exo-aPu^1^/N**. (**b**) The values of the observed rate constants *k*_i_^1^ ([APE1] = 1.0 μM, [**Exo-aPu^1^/N**] = 1.0 μM).

It has been shown earlier [32,39] that the efficiency of a 3′-5′ exonuclease reaction depends on the thermal stability of DNA duplexes. In this paper, we studied the effect of the terminal 3′-base pair on the removal of the first 3′-end aPu nucleotide. For this purpose, duplexes **Exo-aPu^1^/N** containing bases cytosine, thymine, adenine, and guanine opposite aPu (N = C, Т, A, and G) were used. The kinetic traces (Figure 7a) obtained for the interactions of APE1 (1.0 μM) with duplexes **Exo-aPu^1^/N** (1.0 μM) revealed that the rate of aPu removal depends on the opposite base.

The kinetic traces presented in Figure 7a were estimated using Equation (3) where *i* = 2 and the values of the observed rate constants of the first and the second phases *k*_1_^1^ and *k*_2_^1^, respectively, were calculated. Following from the preceding discussion, the rate constant *k*_1_^1^ characterizes DNA binding and detachment of the 3′-aPu nucleotide while *k*_2_^1^ reflects the release of an aPu nucleotide. As presented in Table 1 and Figure 7b, only the rate constant of 3′-end aPu detachment *k*_1_^1^ depends on the opposite base and increases in the order of T < C < A < G. It was shown in Reference [40] that the stability of an aPu pair with one of the natural DNA bases decreases in the same order. In fact, 2-aminopurine forms two hydrogen bonds with thymine or one hydrogen bond with cytosine, but it does not form hydrogen bonds with purine bases. Therefore, our data are consistent with the conclusion that the formation of a catalytically active complex of APE1 proceeds faster with the substrates containing a mismatched purine base opposite of 2-aminopurine, i.e., aPu/G and aPu/А due to the absence of any complementary interactions and destabilization of the 3′ end. The obtained data are in agreement with recent structural data [23] suggesting that APE1 likely removes a matching base through the same mechanism as a mismatched base only at lower efficiency owing to the relative lack of flexibility at the 3′ end. It was shown in this study that the stable C/G base pairing prevents the phosphate backbone from entering the proper registry for cleavage by the APE1 active site. A comparison of structures of the APE1 complex with matching C/G and mismatched C/T substrates reveals that the matched C is shifted 7.5 Å downstream and away from the key catalytic residues.

The observed rate constants of the second phase *k*_2_^1^ expectedly do not depend on the nature of the opposite nucleotide because this phase reflects the aPu nucleotide release from the active site of the enzyme. The calculated value of *k*_2_^1^ for all substrates **Exo-aPu^1^/N** was equal to 0.0034 ± 0.0002 s^−1^ and was very close to the same value determined for an aPu release by analysis involving a set of concentrations of substrate **Exo-aPu^1^/T** (*k*_release_^aPu^ = 0.0038 ± 0.0002 s^−1^, Figure 5b) and the mean rate constant of a release of any 3′-end nucleotide (*k*_release_ = 0.0035 ± 0.0001 s^−1^, Figure 6b).

### 2.4. The Rate of the 3′-End Nucleotide Removal Depends on Its Structure

For the assay of the impact of the 3′-end nucleotide structure on the rate of its removal, duplexes **Exo-aPu^2^-X/N** were used as DNA substrates. These duplexes contained an aPu nucleotide at the second position from the 3′ end, but the first position was occupied by nucleotide **X** and contained either an undamaged base (adenine (A), guanine (G), cytosine (C), or thymine (T)) or a modified base: 8-oxoguanine (oxoG), uracil (U), 5-methylcytosine (5mC), α-anomer of adenosine (αA), or representing the 2-oxymethyl-3-oxy-tetrahydrofurane residue (F) or phosphate (p).

**Figure 8:**
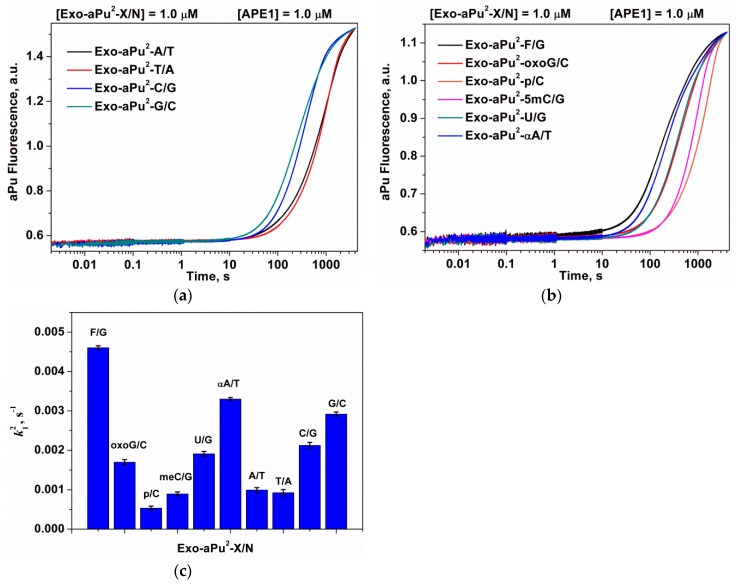
A change in the intensity of aPu in the course of APE1 interaction with substrates **Exo-aPu^2^-X/N** that contain an undamaged (**a**) or damaged (**b**) 3′-end nucleotide. (**c**) The values of the observed rate constants *k*_i_^2^, **X/N**-pair is depicted in figure. [APE1] = 1.0 μM, [**Exo-aPu^1^/N**] = 1.0 μM.

The aPu fluorescence kinetic curves obtained for the case of interaction of APE1 with substrates **Exo-aPu^2^-X/N** are presented in Figure 8a,b. The initial exponential increase of the aPu fluorescence was fitted to Equation (3) to calculate the observed rate constant *k*_1_^2^ (Figure 8c and Table 2). It is evident from these data that *k*_1_^2^ changes in a rather narrow range (0.5–4.6) × 10^−3^ s^−1^.

According to previous analysis, *k*_1_^2^ may characterize a combination of processes of DNA binding, detachment and release of the 3′-X nucleotide, and subsequent detachment of the aPu nucleotide. In the approximation where the binding of different **Exo-aPu^2^-X/N** substrates can be considered the same, the difference in the rate constant *k*_1_^2^ is due to the difference in the rates of the X nucleotide detachment and release from the active site. Moreover, because the nucleotide detachment rate is at least 20-fold higher than the rate of the nucleotide release, it could be concluded that the release of the nucleotide from the active site determines the differences in the observed constants *k*_1_^2^.

The rates of removal of the aPu nucleotide are higher when it is located after the G/C and C/G pairs in comparison with the A/T and T/A pairs. In the case when aPu is located after a damaged X/N pair, the values of *k*_1_^2^ decrease in the following order: F/G ≥ αA/T > oxoG/C = U/G > meC/G ≥ p/C, which indicates the maximal release rate for the abasic nucleotide. For duplexes containing base pairs oxoG/C, U/G, or C/G, the values of *k*_1_^2^ are very close and are in the range (1.7–2.1) × 10^−3^ s^−1^. Otherwise, for the duplex with a G/C base pair, the observed rate constant *k*_1_^2^ is sufficiently higher and equal to 2.9 × 10^−3^ s^−1^. The minimal value of *k*_1_^2^ (5.3 × 10^−4^ s^−1^) was detected for the removal of a 3′-phosphate group in the case of **Exo-aPu^2^-p/C**, which indicates that the 3′-phosphatase activity of APE1 is weaker than its 3′-5′ exonuclease activity.

## 3. Materials and Methods

### 3.1. Protein Expression and Purification

To purify APE1 expressed as a recombinant protein, 1 L of culture (in Luria-Bertani (LB) broth) of the *Escherichia coli* strain Rosetta II(DE3) (Merck KGaA, Darmstadt, Germany) carrying the pET11a-APE1 construct was grown with 50 μg/mL ampicillin at 37 °C until absorbance at 600 nm (A_600_) reached 0.6 to 0.7. APE1 expression was induced overnight with 0.2 mM isopropyl-β-d-thiogalactopyranoside. The method for isolation of wild-type APE1 has been described previously (for more details, see Supplementary Materials: Figure S1) [41]. The protein concentration was measured by the Bradford method [42]. The stock solution was stored at −20 °C.

### 3.2. Oligodeoxyribonucleotides

The sequences of oligodeoxyribonucleotides used in this work are listed in Table 3. The oligodeoxyribonucleotides were synthesized by the standard phosphoramidite method on an ASM-700 synthesizer (BIOSSET, Novosibirsk, Russia) from phosphoramidites purchased from Glen Research (Sterling, VA, USA). α-2′-Deoxyadenosine phosphoramidite was bought from ChemGenes Corp. (Wilmington, MA, USA). Synthetic oligonucleotides were unloaded from the solid support with ammonium hydroxide, according to manufacturer protocols. Deprotected oligonucleotides were purified by HPLC. Concentrations of oligonucleotides were calculated from their absorbance at 260 nm. Oligodeoxyribonucleotide duplexes were prepared by annealing modified and complementary strands at a 1:1 molar ratio. Shorthands of DNA duplexes denote the number of position for aPu residue from the 3′ end in the short oligonucleotide and letter “N”, which correspond to the T, A, G, or C nucleotide placed opposite to the 3′ end of the short oligonucleotide in the 5′ dangling oligonucleotide.

### 3.3. Stopped-Flow Experiments

These experiments were conducted essentially as described previously [43,44,45]. An SX.18MV stopped-flow spectrometer (Applied Photophysics, Leatherhead, UK) fitted with a 150 W Xe arc lamp and a 2 mm path length optical cell was employed. The dead time of the instrument was 1.4 ms. The excitation wavelength was 310 nm for the aPu fluorescent dye. The emission was monitored using a long-pass wavelength filter (Corion, Franklin, MA, USA) at 370 nm. APE1 was placed in one of the instrument′s syringes and rapidly mixed with the substrate in another syringe. The reported concentrations of reactants are those in the reaction chamber after mixing. Typically, each trace shown in the figures is the average of four or more fluorescence traces recorded in individual experiments. In the figures, if necessary for better presentation, the curves were manually moved apart. All the experiments were carried out in a buffer consisting of 50 mM Tris-HCl pH 7.5, 50 mM KCl, 5 mM MgCl_2_, 1 mM EDTA, 1 mM DTT, and 7% glycerol (*v*/*v*) at 37 °C.

### 3.4. Product Analysis

To analyze the products formed by APE1, the substrates were 5′-^32^P-labeled with phage T4 polynucleotide kinase and γ-^32^P-ATP. The reaction was carried out under the conditions described above. Cleavage of the substrate was initiated by the addition of APE1. Aliquots (2 μL) of the reaction mixture were taken at certain time intervals, immediately quenched with 3 μL of a gel-loading dye containing 7 M urea and 50 mM EDTA, and loaded on a 20% (*w*/*v*) polyacrylamide gel containing 7 M urea. Disappearance of the substrate and formation of the products were analyzed by autoradiography and quantified by scanning densitometry in the Gel-Pro Analyzer software, v.4.0 (Media Cybernetics, Rockville, MD, USA).

### 3.5. Kinetic Data Analysis

Stopped-flow kinetic traces were fitted to Equation (3) by a nonlinear regression procedure in the Origin software (OriginLab Corp., Northampton, MA, USA).

(3) $$F_{c}\;=\;F_{b}+\overset{N}{\underset{i=0\;}{\sum }}A_{i}\;\times \;exp\;(-k_{i}\;\times t)$$

where *F_c_* is the observed fluorescence intensity of aPu, *F_b_* indicates background fluorescence, *A_i_* denotes fluorescence parameters, *k_i_* is the observed rate constant, and *t* is the reaction time.

## 4. Conclusions

In general, our findings revealed that in a 3′-5′ exonuclease reaction, removal of the first 3′-terminal nucleotide proceeds significantly faster (approximately 35-fold) than the removal of subsequent nucleotides located at the next positions from the 3′ end. This finding indicates that catalysis during the first enzymatic turnover is more rapid than in the subsequent enzymatic cycles, which supports the appearance of the rate-limiting step after catalytic hydrolysis of the phosphodiester bond. It is reasonable to conclude that the deceleration of the removal rate for the subsequent nucleotides in comparison with the first one is caused by a slow release of the detached nucleotide from the active site of the enzyme. It was shown that the rate of aPu removal from the 3′ end depends inversely on the stability of the aPu pair with undamaged DNA bases. The obtained kinetic data show that the effect of pair stability is relatively moderate and a difference in the observed rate constant does not exceed five-fold, which suggests that formation of the catalytic complex with any pairs at the 3′ end proceeds rapidly and efficiently. A comparison of various undamaged and damaged 3′-end nucleotides revealed that the rate of the release of the detached nucleotide from the active site of the enzyme varies ≤10-fold with maximum and minimum values corresponding to an abasic nucleotide and phosphate group, respectively. Most likely this variation is associated with differences in the nucleotide structure. Taken together, our results are suggestive of a kinetic mechanism of the 3′-5′ exonuclease reaction and indicate that the release of a detached nucleotide from the active site of APE1 is the rate-limiting step of APE1 3′-5′ exonuclease activity.

## Figures and Tables

**Table 1:** The rate constants of the interaction of APE1 with substrates **Exo-aPu^1^/N**.

DNA	DNA Binding and aPu Detaching, *k*_1_^1^, s^−1^	aPu Releasing, *k*_2_^1^, s^−1^
**Exo-aPu^1^/G**	0.13 ± 0.02	0.0035 ± 0.0002
**Exo-aPu^1^/A**	0.075 ± 0.001	0.0035 ± 0.0002
**Exo-aPu^1^/C**	0.050 ± 0.001	0.0031 ± 0.0002
**Exo-aPu^1^/T**	0.040 ± 0.001	0.0034 ± 0.0002

**Table 2:** The rate constants *k*_1_^2^ of the interaction of APE1 with substrates **Exo-aPu^2^-X/N**.

X/N Nature	*k*_1_^2^, s^−1^
F/G	(4.6 ± 0.05) × 10^−3^
oxoG/C	(1.7 ± 0.07) × 10^−3^
p/C	(5.3 ± 0.6) × 10^−4^
5mC/G	(8.9 ± 0.5) × 10^−4^
U/G	(1.9 ± 0.06) × 10^−3^
αA/T	(3.3 ± 0.04) × 10^−3^
A/T	(9.9 ± 0.6) × 10^−4^
T/A	(9.2 ± 0.5) × 10^−4^
C/G	(2.1 ± 0.07) × 10^−3^
G/C	(2.9 ± 0.05) × 10^−3^

**Table 3:** DNA duplexes used as substrates and ligands of APE1.

Shorthand	Sequence
**Exo-aPu^1^/N** N = A, G, C, T	5′-CAGCTCTGTACGTG(**aPu**)-3′ 3′-GTCGAGACATGCAC **N** CGTCACCACTGTG-5′
**Exo-aPu^2^/T**	5′-CAGCTCTGTACGT(**aPu**)A-3′ 3′-GTCGAGACATGCA C **T**CGTCACCACTGTG-5′
**Exo-aPu^4^/T**	5′-CAGCTCTGTAC(**aPu**)TGA-3′ 3′-GTCGAGACATG C AC**T**CGTCACCACTGTG-5′
**Exo-aPu^6^/T**	5′-CAGCTCTGT(**aPu**)CGTGA-3′ 3′-GTCGAGACA T GCAC**T**CGTCACCACTGTG-5′
**Exo-aPu^2^-X/N** N = A, G, C, T X = F, G, C, T, oxoG, U, 5mC, p, αA	5′-CAGCTCTGTACGT(**aPu**)**X**-3′ 3′-GTCGAGACATGCA C **N**CGTCACCACTGTG-5′

## Supplementary Materials

The following are available online: Figure S1.

## References

[1] Li M., Wilson D.M. (2014). Human apurinic/apyrimidinic endonuclease 1. Antioxid. Redox. Signal..

[2] Mol C.D., Parikh S.S., Putnam C.D., Lo T.P., Tainer J.A. (1999). DNA repair mechanisms for the recognition and removal of damaged DNA bases. Annu. Rev. Biophys. Biomol. Struct..

[3] David S.S., Williams S.D. (1998). Chemistry of glycosylases and endonucleases involved in base-excision repair. Chem. Rev..

[4] Demple B., Sung J.-S. (2005). Molecular and biological roles of Ape1 protein in mammalian base excision repair. DNA Repair.

[5] Dyrkheeva N.S., Khodyreva S.N., Lavrik O.I. (2007). Multifunctional human apurinic/apyrimidinic endonuclease 1: Role of additional functions. Mol. Biol..

[6] Parsons J.L., Dianova I.I., Dianov G.L. (2004). APE1 is the major 3′-phosphoglycolate activity in human cell extracts. Nucleic Acids Res..

[7] Suh D., Wilson D.M., Povirk L.F. (1997). 3′-Phosphodiesterase activity of human apurinic/apyrimidinic endonuclease at DNA double-strand break ends. Nucleic Acids Res..

[8] Barnes T., Kim W.C., Mantha A.K., Kim S.E., Izumi T., Mitra S., Lee C.H. (2009). Identification of Apurinic/apyrimidinic endonuclease 1 (APE1) as the endoribonuclease that cleaves c-myc mRNA. Nucleic Acids Res..

[9] Daviet S., Couve-Privat S., Gros L., Shinozuka K., Ide H., Saparbaev M., Ishchenko A.A. (2007). Major oxidative products of cytosine are substrates for the nucleotide incision repair pathway. DNA Repair.

[10] Gros L., Ishchenko A.A., Ide H., Elder R.H., Saparbaev M.K. (2004). The major human AP endonuclease (Ape1) is involved in the nucleotide incision repair pathway. Nucleic Acids Res..

[11] Prorok P., Saint-Pierre C., Gasparutto D., Fedorova O.S., Ishchenko A.A., Leh H., Buckle M., Tudek B., Saparbaev M. (2012). Highly mutagenic exocyclic DNA adducts are substrates for the human nucleotide incision repair pathway. PLoS ONE.

[12] Christov P.P., Banerjee S., Stone M.P., Rizzo C.J. (2010). Selective Incision of the alpha-*N*-Methyl-Formamidopyrimidine Anomer by Escherichia coli Endonuclease IV. J. Nucleic Acids.

[13] Vrouwe M.G., Pines A., Overmeer R.M., Hanada K., Mullenders L.H. (2011). UV-induced photolesions elicit ATR-kinase-dependent signaling in non-cycling cells through nucleotide excision repair-dependent and -independent pathways. J. Cell Sci..

[14] Guliaev A.B., Hang B., Singer B. (2004). Structural insights by molecular dynamics simulations into specificity of the major human AP endonuclease toward the benzene-derived DNA adduct, pBQ-C. Nucleic Acids Res..

[15] Prorok P., Alili D., Saint-Pierre C., Gasparutto D., Zharkov D.O., Ishchenko A.A., Tudek B., Saparbaev M.K. (2013). Uracil in duplex DNA is a substrate for the nucleotide incision repair pathway in human cells. Proc. Natl. Acad. Sci. USA.

[16] Ischenko A.A., Saparbaev M.K. (2002). Alternative nucleotide incision repair pathway for oxidative DNA damage. Nature.

[17] Gorman M.A., Morera S., Rothwell D.G., de La Fortelle E., Mol C.D., Tainer J.A., Hickson I.D., Freemont P.S. (1997). The crystal structure of the human DNA repair endonuclease HAP1 suggests the recognition of extra-helical deoxyribose at DNA abasic sites. EMBO J..

[18] Beernink P.T., Segelke B.W., Hadi M.Z., Erzberger J.P., Wilson D.M., Rupp B. (2001). Two divalent metal ions in the active site of a new crystal form of human apurinic/apyrimidinic endonuclease, Ape1: Implications for the catalytic mechanism. J. Mol. Biol..

[19] Manvilla B.A., Pozharski E., Toth E.A., Drohat A.C. (2013). Structure of human apurinic/apyrimidinic endonuclease 1 with the essential Mg^2+^ cofactor. Acta Crystallogr. D Biol. Crystallogr..

[20] Mol C.D., Izumi T., Mitra S., Tainer J.A. (2000). DNA-bound structures and mutants reveal abasic DNA binding by APE1 and DNA repair coordination. Nature.

[21] Mol C.D., Hosfield D.J., Tainer J.A. (2000). Abasic site recognition by two apurinic/apyrimidinic endonuclease families in DNA base excision repair: The 3′ ends justify the means. Mutat. Res..

[22] Tsutakawa S.E., Shin D.S., Mol C.D., Izumi T., Arvai A.S., Mantha A.K., Szczesny B., Ivanov I.N., Hosfield D.J., Maiti B. (2013). Conserved structural chemistry for incision activity in structurally non-homologous apurinic/apyrimidinic endonuclease APE1 and endonuclease IV DNA repair enzymes. J. Biol. Chem..

[23] Whitaker A.M., Flynn T.S., Freudenthal B.D. (2018). Molecular snapshots of APE1 proofreading mismatches and removing DNA damage. Nat. Commun..

[24] Hadi M.Z., Ginalski K., Nguyen L.H., Wilson D.M. (2002). Determinants in nuclease specificity of Ape1 and Ape2, human homologues of Escherichia coli exonuclease III. J. Mol. Biol..

[25] Mundle S.T., Fattal M.H., Melo L.F., Coriolan J.D., O’Regan N.E., Strauss P.R. (2004). Novel role of tyrosine in catalysis by human AP endonuclease 1. DNA Repair.

[26] Mundle S.T., Delaney J.C., Essigmann J.M., Strauss P.R. (2009). Enzymatic mechanism of human apurinic/apyrimidinic endonuclease against a THF AP site model substrate. Biochemistry.

[27] Timofeyeva N.A., Koval V.V., Knorre D.G., Zharkov D.O., Saparbaev M.K., Ishchenko A.A., Fedorova O.S. (2009). Conformational dynamics of human AP endonuclease in base excision and nucleotide incision repair pathways. J. Biomol. Struct. Dyn..

[28] Kanazhevskaya L.Y., Koval V.V., Zharkov D.O., Strauss P.R., Fedorova O.S. (2010). Conformational transitions in human AP endonuclease 1 and its active site mutant during abasic site repair. Biochemistry.

[29] Kanazhevskaya L.Y., Koval V.V., Vorobjev Y.N., Fedorova O.S. (2012). Conformational dynamics of abasic DNA upon interactions with AP endonuclease 1 revealed by stopped-flow fluorescence analysis. Biochemistry.

[30] Miroshnikova A.D., Kuznetsova A.A., Vorobjev Y.N., Kuznetsov N.A., Fedorova O.S. (2016). Effects of mono- and divalent metal ions on DNA binding and catalysis of human apurinic/apyrimidinic endonuclease 1. Mol. Biosyst..

[31] Miroshnikova A.D., Kuznetsova A.A., Kuznetsov N.A., Fedorova O.S. (2016). Thermodynamics of Damaged DNA Binding and Catalysis by Human AP Endonuclease 1. Acta Nat..

[32] Dyrkheeva N.S., Lomzov A.A., Pyshnyi D.V., Khodyreva S.N., Lavrik O.I. (2006). Efficiency of exonucleolytic action of apurinic/apyrimidinic endonuclease 1 towards matched and mismatched dNMP at the 3′ terminus of different oligomeric DNA structures correlates with thermal stability of DNA duplexes. Biochim. Biophys. Acta.

[33] Lebedeva N.A., Khodyreva S.N., Favre A., Lavrik O.I. (2003). AP endonuclease 1 has no biologically significant 3′→5′-exonuclease activity. Biochem. Biophys. Res. Commun..

[34] Wilson D.M. (2003). Properties of and substrate determinants for the exonuclease activity of human apurinic endonuclease Ape1. J. Mol. Biol..

[35] Jean J.M., Hall K.B. (2001). 2-Aminopurine fluorescence quenching and lifetimes: Role of base stacking. Proc. Natl. Acad. Sci. USA.

[36] Rachofsky E.L., Osman R., Ross J.B.A. (2001). Probing structure and dynamics of DNA with 2-aminopurine: Effects of local environment on fluorescence. Biochemistry.

[37] Hardman S.J., Thompson K.C. (2006). Influence of base stacking and hydrogen bonding on the fluorescence of 2-aminopurine and pyrrolocytosine in nucleic acids. Biochemistry.

[38] Atkins P., Paula J. (2006). Atkins’ Physical Chemistry.

[39] Chou K.M., Cheng Y.C. (2002). An exonucleolytic activity of human apurinic/apyrimidinic endonuclease on 3’ mispaired DNA. Nature.

[40] Law S.M., Eritja R., Goodman M.F., Breslauer K.J. (1996). Spectroscopic and calorimetric characterizations of DNA duplexes containing 2-aminopurine. Biochemistry.

[41] Kuznetsova A.A., Kuznetsov N.A., Ishchenko A.A., Saparbaev M.K., Fedorova O.S. (2014). Pre-steady-state fluorescence analysis of damaged DNA transfer from human DNA glycosylases to AP endonuclease APE1. Biochim. Biophys. Acta.

[42] Bradford M.M. (1976). A rapid and sensitive method for the quantitation of microgram quantities of protein utilizing the principle of protein-dye binding. Anal. Biochem.

[43] Kuznetsov N.A., Kuznetsova A.A., Vorobjev Y.N., Krasnoperov L.N., Fedorova O.S. (2014). Thermodynamics of the DNA damage repair steps of human 8-oxoguanine DNA glycosylase. PLoS ONE.

[44] Kuznetsova A.A., Kuznetsov N.A., Vorobjev Y.N., Barthes N.P., Michel B.Y., Burger A., Fedorova O.S. (2014). New Environment-Sensitive Multichannel DNA Fluorescent Label for Investigation of the Protein-DNA Interactions. PLoS ONE.

[45] Kuznetsova A.A., Iakovlev D.A., Misovets I.V., Ishchenko A.A., Saparbaev M.K., Kuznetsov N.A., Fedorova O.S. (2017). Pre-steady-state kinetic analysis of damage recognition by human single-strand selective monofunctional uracil-DNA glycosylase SMUG1. Mol. Biosyst..

