# Supplementary material for: Kinetic Features of 3′-5′ Exonuclease Activity of Human AP-Endonuclease APE1
Source: Molecules. 2018 Aug 21;23(9):2101. doi: 10.3390/molecules23092101 (PMC6225374; doi:10.3390/molecules23092101)
Supplement: Supplementary file 1 [file molecules-23-02101-s001.pdf]

Supplementary Material

Kinetic Features of 3′-5′ Exonuclease Activity of Human AP-Endonuclease APE1

Alexandra A. Kuznetsova [1], Olga S. Fedorova [1,2,]* and Nikita A. Kuznetsov [1,2,]*

1Institute of Chemical Biology and Fundamental Medicine, Siberian Branch of the Russian Academy of Sciences, Novosibirsk, 630090, Russia

[2]Department of Natural Sciences, Novosibirsk State University, Novosibirsk 630090, Russia

* Correspondence: fedorova@niboch.nsc.ru (O.S.F.); nikita.kuznetsov@niboch.nsc.ru (N.A.K.); Tel.: +7 383-363-5175 (O.S.F.); +7 383-363-5174(N.A.K.); Fax: +7 383-363-5153 (O.S.F. and N.A.K)

Protein purification

The cell lysate was centrifuged (40,000×g, 40 min), and the supernatant was loaded onto column I (Q-Sepharose Fast Flow, Amersham Biosciences, Sweden) with subsequent washing with buffer solution I (20 mM HEPES-NaOH, pH 7.8) containing 100 mM NaCl. Fractions containing the protein were collected and loaded onto column II (HiTrap-Heparin™ Amersham Biosciences, Sweden) in buffer solution II (20 mM HEPES-NaOH, pH 7.8) containing 40 mM NaCl. Chromatography was run in buffer solution II and a linear gradient of 40 → 600 mM NaCl. The solution's absorbance was detected at a wavelength of 280 nm and 292 nm (Figure S1A). The protein purity was determined by gel electrophoresis (Figure S1B). Fraction 2 (see lane 7) was used in the work.

A

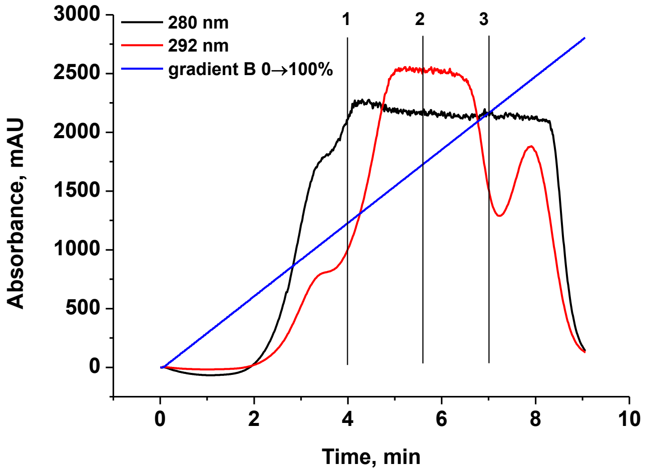

B

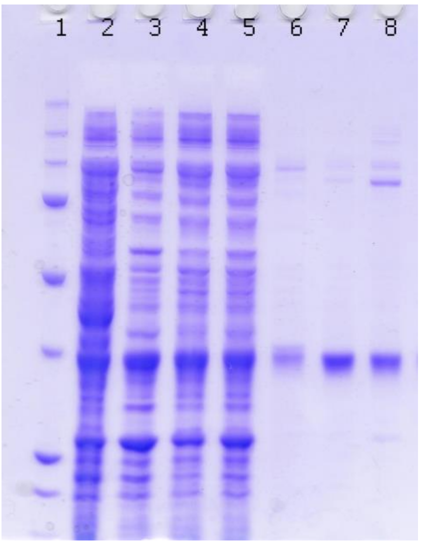

Figure S1. APE1 purification. (A) Proteins elution from Heparin column in a 40–600 mM NaCl gradient. (B) PAGE analysis of APE1 purification: 1, protein marker (250, 150,100, 75, 50, 37, 25, 20 kDa); 2, total cell lysate; 3, flow through Q sepharose (last drop); 4, flow through Q sepharose (for loading on Heparin); 5, flow through Heparin; 6, fraction 1; 7, fraction 2; 8, fraction 3.
